# Nighttime screen use, sleep quality, and smartphone addiction symptoms among medical students: an international cross-sectional study

**DOI:** 10.3389/fpsyt.2026.1735186

**Published:** 2026-02-06

**Authors:** Lukas Liebig, Erika Balogh, Béla Birkás, Nóra Faubl, Erika Zelko, Willy Gräfe, Sophie Pieper, Mandy Gottschall, Henna Riemenschneider

**Affiliations:** 1 Department of General Practice, Faculty of Medicine Carl Gustav Carus, Dresden University of Technology, Dresden, Germany; 2 Department of Public Health Medicine, University of Pécs, Medical School, Pécs, Hungary; 3 Department of Behavioural Sciences, University of Pécs, Medical School, Pécs, Hungary; 4 Institute of General Practice, Johannes Kepler University Linz, Linz, Austria

**Keywords:** cross-sectional study, medical students, nighttime screen use, Pittsburgh Sleep Quality Index (PSQI), sleep quality, smartphone addiction

## Abstract

**Background:**

Nighttime screen use is associated with poor sleep and may represent an emerging public health concern. Medical students are a vulnerable group for harmful health behaviour due to demanding studies, while also playing a key role in future health promotion. The study examined the prevalence of nighttime screen use among medical students in different countries and explored associations with sleep and smartphone addiction symptoms.

**Methods:**

The Medical Student Health Survey, an international cross-sectional study, was conducted at four sites in Germany (GER), Austria (AU), Hungary (HU), and Japan (JA). Nighttime screen use was assessed via five self-developed items, sleep via Pittsburgh Sleep Quality Index (PSQI), and smartphone addiction symptoms via Smartphone Addiction Scale–Short Version (SAS-SV). Group differences were tested using chi-square, Welch’s t-test, Z-test, and Kruskal–Wallis test (Bonferroni-adjusted), and associations were evaluated using Kendall’s tau-b and a multivariable binary logistic regression model.

**Results:**

Data from n = 1,262 medical students (mean age 21–24, 56%–73% female) were analysed. The median pre-bedtime screen use was 30 minutes, and the median end of use was between 5 and 10 minutes before bedtime. Approximately 96%–98% used a screen within 1 hour of bedtime; 69%–89% used a smartphone/tablet. SAS-SV scores ranged from M = 23.2 (HU) to M = 27.0 (JA), and the prevalence of smartphone addiction ranged from 12.6% (GER) to 27.9% (JA). PSQI scores ranged from M = 4.33 (JA) to M = 5.22 (HU), and prevalence of poor sleep quality ranged from 25% (JA) to 40.7% (HU). Prolonged pre-bedtime screen use was associated with increased sleep latency (τ = 0.106, p <.001), shorter distance between the end of screen use and bedtime, and increased SAS-SV scores (τ = −0.150, p <.001). Screen use after waking up at night was associated with poorer sleep quality (τ = 0.174, p <.001), poorer outcome in five of seven PSQI subscales (all τ > 0.1, p <.001), and increased SAS-SV scores (τ = 0.165, p <.001). Sleep disturbance by a device was associated with poorer sleep quality (τ = 0.170, p <.001), poorer outcome in three of seven PSQI subscales (all τ > 0.1, p <.001), and increased SAS-SV scores (τ = 0.135, p <.001). In the logistic regression, screen use after nocturnal awakenings and device-related sleep disturbance remained independently associated with poor sleep quality (all p ≤.04), whereas smartphone addiction symptoms showed no independent association.

**Conclusions:**

The findings highlight the need for targeted interventions to promote health-conscious screen use among medical students.

## Introduction

Medical studies are characterised by a high learning workload, time pressure, and continuous demands on students, which are accompanied by psychological distress (1). Global meta-analyses reveal high prevalence rates of depression (27.2%) (2), burnout (37.2%) (3), and anxiety disorders (33.8%) (4) among medical students. Indeed, psychological health is important for one’s own well-being and in order to fulfil the demands of studying. A prerequisite for psychological health is sufficient sleep and good sleep quality (5–7). A recent international meta-analysis reports (8) that over half of all medical students experience poor sleep quality.

On a national level, screen time among medical students almost doubled within the last 10 years—both for screen time spent for study/work reasons (2.74 hours in 2016 to 5.23 hours in 2024) and for leisure reasons (1.25 hours in 2018 to 2.46 hours in 2024) (not yet published, Liebig et al., data from 2014 to 2024). In a companion study among medical students (9), it was shown that screen time spent during the day is associated with a later bedtime (r = 0.213, p = .001) or shorter sleep duration (r = −0.108, p <.015). Evidence on screen time and sleep quality, however, remains mixed: whereas some studies found no significant relationship (p = .1, 9; p = .2, 10), others (11, 12) demonstrated clear negative associations (p <.001 each). These mixed epidemiological results may be partly explained by the fact that most studies examined overall screen time rather than specifically focusing on evening and nighttime use. According to the model of Cain and Gradisar (13), evening screen time may interfere with sleep through three pathways: 1) sleep displacement (a chronological shift in bedtime), 2) psychophysiological arousal (heightened alertness due to screen-based activities), and 3) suppression of melatonin by blue-light exposure. Therefore, the National Sleep Foundation (14) recommends avoiding screen use at least 1 hour before going to bed. In line with this framework and existing studies (15, 16), it seems plausible that longer evening screen exposure and a shorter interval between ending screen use and bedtime are related to poorer sleep outcomes (e.g., longer sleep-onset latency, later bedtime, shorter total sleep time).

In addition to evening use, further sleep-compromising factors may arise from the omnipresence of smartphones among adolescents and young adults. For example, the device’s mere presence (or device-related cues) can foster phone use (17, 18), which may make media desires more frequent and harder to resist (19). Such urges are reflected in measures of smartphone addiction symptoms like the Smartphone Addiction Scale. Although there is currently no formal clinical diagnosis of “smartphone addiction”, these symptoms are characterised by reduced self-control over smartphone use and may accumulate to long-term detrimental impacts on normal life routines (20). In the context of sleep, this lack may increase evening screen use in the short term and, in the long term, make it harder to establish and maintain a healthy sleep routine. A recent meta-analysis (21) (primarily based on Asian student populations) indicates that smartphone addiction affects approximately 40% of medical students.

Moreover, sleep at night can be disturbed both unintentionally (by sounds and light) and intentionally (by consciously checking messages) (22, 23). As a result, sleep is interrupted, important sleep phases may be disrupted, and sleep quality may deteriorate (24). The long-term consequences of sleep disturbance in adolescents include poorer mental health, lower academic performance, and an increased risk of substance use (25).

Analysis of the interaction between sleep and nighttime screen use (defined here as including both evening and late-night screen use) is not only important for the health and academic performance of medical students. As future physicians, medical students’ health habits shape the content (26) and the quality (27) of the lifestyle counselling they provide, thereby influencing patient care. Nevertheless, unfavourable health behaviours are common among medical students (28–30) and practising clinicians (31, 32), with potential effects on care quality and the resilience of the health system. Furthermore, the medical curriculum is marked by high demands and digital workloads. In line with the path-dependency hypothesis (33), behaviours established during training may persist into adulthood; therefore, it is important to identify new types of risk behaviour and, if necessary, raise awareness early.

Based on existing frameworks and prior findings, this study aims to 1) describe the prevalence and patterns of nighttime screen use among medical students and 2) examine how nighttime screen use and smartphone addiction symptoms are associated with sleep quality and sleep behaviour. We hypothesised that longer pre-bedtime screen exposure, a shorter interval between ending screen use and bedtime, and greater nighttime device-related sleep disruption would be associated with poorer sleep outcomes. We further hypothesised that higher smartphone addiction symptoms would be associated with poorer sleep outcomes and more pronounced nighttime screen use.

## Methods

To ensure standardised reporting of key methodological aspects for web-based surveys, the study followed the CHERRIES checklist. Accordingly, we described core procedures (e.g., ethics, development, and administration) in the Methods section, while the completed checklist is provided in **Supplementary File 1**.

### Survey design

Data from enrolled medical students were collected online as part of the cross-sectional, international multicentre study “Medical Student Health Survey” (MSHS). The aim of the MSHS is to assess current trends in health status, health behaviour, and speciality preferences among medical students in order to inform the development of preventive services and the planning of healthcare structures. The MSHS study, which takes place biennially, was conducted in 2024 as a collaboration from the Department of General Practice, Faculty of Medicine and University Hospital Carl Gustav Carus, TUD Dresden University of Technology, Germany (GER); the Institute of General Practice at the Johannes Kepler University Linz, Austria (AU); the Department of Public Health Medicine and the Department of Behavioural Sciences at the Faculty of Medicine of the University of Pécs, Hungary (HU); and the Department of Global Medical Research Promotion, Shinshu University Graduate School of Medicine, Matsumoto, Japan (JA). Thus, the study sites were Dresden (GER), Linz (AU), Pécs (HU), and Matsumoto (JA).

### Ethics and informed consent

The study was registered by the ethics committee at the respective study sites via the following codes: Dresden (EK15012014), Linz (EK1166/2023), Pécs (BMEÜ/2448-1/2022/EKU), and Matsumoto (6176). Before accessing the first survey page, participants viewed an online information sheet outlining the study purpose, voluntariness, data handling, potential risks/benefits, and contact details. They gave informed consent by ticking an “I agree” checkbox. Withdrawal was possible by e-mailing the study team and quoting the optional pseudonymisation code displayed on the debriefing page. The corresponding records were then permanently deleted. No incentives were offered, and non-participation carried no penalties. The survey used the LimeSurvey tool from LimeSurvey GmbH (provided by Bildungsportal Sachsen GmbH). Among other things, LimeSurvey stores a truncated (anonymised) IP address of the respondent’s device together with cookies, which allows participants to resume an interrupted session and prevents multiple submissions from the same user. There was an optional indication of a pseudonymisation code at the end of the study, which was voluntary. Survey responses were saved on an encrypted university server in Germany. No direct identifiers (name, e-mail, and matriculation number) were collected. All procedures adhered to the EU General Data Protection Regulation (GDPR; 2016/679).

### Development, measuring instruments, and pre-testing

The MSHS has been administered annually since 2014 and is a broad survey instrument covering multiple health domains, with different analyses and publications emerging from its various modules. The survey combines validated scales with self-developed items that are refined each wave through qualitative and psychometric feedback. The 2024 instrument comprised sociodemographic questions, four health-related domains—i) health status and behaviour (including sleep and media use), ii) alcohol consumption, iii) tobacco use, and iv) mental health—and items on speciality preference. The present study focuses specifically on sleep, nighttime screen use, and problematic smartphone use.

Nighttime screen use, which includes both evening and late-night screen use, was operationalised as pre-bedtime screen exposure (duration and timing relative to bedtime) and nighttime device-related behaviours (screen use after nocturnal awakenings and device-related sleep disturbance). Because no validated instrument exists to assess nighttime screen use, five self-developed items were created. The items were informed by theoretical and empirical work on mechanisms linking nighttime screen exposure to sleep (e.g., displacement, arousal, and light-related effects) and by prior research on nocturnal awakenings and device-related sleep disruption (13, 22, 23). The items underwent cognitive pre-testing (think-aloud) with 15 participants (medical students, clinicians, and scientific staff). The pre-tests focused on comprehension and response processes, and wording was iteratively refined to maximise face validity. The items covered the following: 1) duration of screen use before bedtime (minutes), 2) cessation time before sleep (minutes), 3) device type, 4) frequency of screen use after nocturnal awakenings, and 5) sleep interruptions by device. The five items were analysed as separate behavioural indicators (rather than as a unidimensional scale). Therefore, internal consistency indices and factor-analytic validation were not applicable. Sleep quality was measured using the Pittsburgh Sleep Quality Index (PSQI) (34, 35), and smartphone addiction symptoms were assessed using a translated version of the Smartphone Addiction Scale–Short Version (SAS-SV) (36). Both are cross-culturally validated and widely used, allowing direct comparison with international research. The translated version of the SAS-SV in the German language had already been tested in a previous study (not yet published, Liebig et al., data from 2022) and showed good internal consistency among medical students (α = .82). The final English version of the nighttime screen use items, PSQI, and SAS-SV are provided in **Supplementary File 2**.

Translation followed a peer-reviewed forward–backward procedure. For each target language (English, Hungarian, and Japanese), two native-speaking bilinguals with a health-science background produced independent forward translations and reconciled them. An independent bilingual with advanced German proficiency back-translated the draft. An expert panel compared the back-translation with the German source, resolved discrepancies, and adapted cultural references. The near-final version was cognitively debriefed by ≥5 native speakers per language (including ≥1 medical student), and minor wording changes were incorporated. The LimeSurvey implementation was beta-tested in every language by ≥5 research-staff members to verify skip logic and device compatibility, with an average completion time of ~15 minutes.

### Recruitment and eligibility

A closed, voluntary, pseudonymous web survey was administered. The survey link was distributed only to enrolled medical students through faculty e-mail newsletters, posts in student-run social-media groups, and brief announcements during lectures and seminars. There were no additional inclusion or exclusion criteria. Participants could complete the questionnaire in German, Hungarian, Japanese, or English.

### Survey administration

The questionnaire was delivered in LimeSurvey version 2.50+ with conditional branching: follow-up questions appeared only when a preceding response made them relevant—for example, the detailed tobacco section was shown only to students who reported recent consumption. No question was technically forced, no randomisation was applied, and LimeSurvey performed no completeness checks. Respondents could navigate backwards at any time to revise earlier answers. Because of the branching logic, the total number of items—and items per page—varied between participants. To prevent multiple submissions, LimeSurvey stored a session cookie and an anonymised IP address. The survey periods were site-specific: Hungary (Pécs), February–May 2024; Austria (Linz), March–July 2024; Germany (Dresden), April–July 2024; and Japan (Matsumoto), June–July 2024.

### Cofactors

As various factors can influence sleep behaviour and screen use (9, 37, 38), information on financial situation, housing situation, physical activity, and partnership was taken into account in addition to sociodemographic data (age, gender, and semester/year of study). Whether students were in a stable, long-term relationship was recorded using the answers “yes” or “no”, while the housing situation was categorised as “living alone” or “not living alone”. Financial problems (Likert scale 1–5) were dichotomised into “no” (1–2) and “existing financial problems” (3–5). European students were assigned to the “clinical” (as opposed to “preclinical”) study phase in the fifth semester, and Japanese students in the fifth year of study. Students were considered physically active according to the WHO guideline (39) if they were active for at least 150 minutes per week at moderate intensity or at least 75 minutes per week at high intensity.

### Statistical analyses

Analyses were conducted as complete-case analyses. Participants with missing data on nighttime screen use, PSQI, or SAS-SV were excluded. No weighting or propensity score adjustment was applied. No analysis of atypical timestamps was performed, as the survey allowed respondents to pause and resume completion at a later time point.

To ensure a statistically sound analysis, the response options for nighttime screen use were combined as never, less than once a week, and once to several times a week. The components contained in the PSQI (subjective sleep quality, sleep latency, sleep duration, sleep efficiency, sleep disturbance, use of sleep medication, and daytime dysfunction) were determined for each student, and a PSQI total score (0–21) was calculated to assess sleep quality. A global PSQI score >5 indicated poor sleep quality. According to the authors of the SAS-SV (36), a total score (10–60) was formed and used to determine smartphone addiction in accordance with the threshold values (31 for men and 33 for women). If no or diverse gender information was provided, a SAS-SV cut-off of 33 was used to determine smartphone addiction.

The statistical analyses were carried out using R-Studio 4.2.3 and IBM SPSS 29.0-30.0. The data were tested for normal distribution using the Shapiro–Wilk test and the Kolmogorov–Smirnov test. Internal consistency was determined using Cronbach’s alpha. Group-specific differences in mean values of non-normally distributed metric data were tested for n = 2 groups with a Welch’s t-test, which is robust to violations of homoscedasticity and performs well in larger samples despite deviations from normality (40) and for n > 2 groups with a Kruskal–Wallis test for significance (p <.05). Group-specific differences in proportions were tested for significance with a significant chi-square test via Z-test or Fisher’s exact test (n < 10). Pairwise comparisons between study sites were Bonferroni-corrected. Kendall’s tau-b correlation coefficients were calculated to determine the associations between nighttime screen use, sleep behaviour, and smartphone use. An adjusted multivariable binary logistic regression model was conducted with poor sleep quality (PSQI > 5) as the dependent variable. Predictors included sociodemographic variables (including study site), nighttime screen use, and smartphone addiction symptoms (z-standardised SAS-SV). Multicollinearity was assessed using Generalized Variance Inflation Factor (GVIF), and linearity in the logit for continuous predictors was checked using the Box–Tidwell approach. The model fit was evaluated using the Hosmer–Lemeshow test and Nagelkerke’s R^2^.

## Results

### Study population

In Dresden (GER), n = 481 of n = 1,997 enrolled students completed the questionnaire (24.1%). In Linz (AU), n = 199 of n = 1,554 (12.8%) participated; in Pécs (HU), n = 989 of n = 2,710 (36.6%); and in Matsumoto (JA), n = 199 of n = 742 (26.8%). Only data from those students who provided complete information in the PSQI and the SAS-SV, and with regard to nighttime screen use, were included. The final study sample comprised n = 1,262 medical students (GER, n = 301; AU, n = 137; HU, n = 720; and JA, n = 104). The mean age of the study population was 24.0 (SD 3.7) years in Germany, 24.0 (SD 3.3) years in Austria, 22.2 (SD 3.0) years in Hungary, and 21.0 (SD 3.1) years in Japan. Most respondents were female, varying from 56% in Japan to 73% in Germany (see **Table 1** for sociodemographic data). Compared with the excluded respondents, the included participants differed in study site distribution, age, gender, and housing situation (all p <.01, largest Cohen’s d = 0.17), whereas study phase, financial situation, and physical activity did not differ; full results including effect sizes are provided in **Supplementary File 3**.

**Table 1:** Sociodemographic characteristics of the study sites.

Characteristics	GER (n = 301)	AU (n = 137)	HU (n = 720)	JA (n = 104)
Age, M (SD)	24.0 (3.7)	24.0 (3.3)	22.2 (3.0)	21.0 (3.1)
Gender, n (%)				
Male Female Not specified, diverse	80 (26.6) 219 (72.8) 2 (.6)	46 (33.6) 91 (66.4) -	256 (35.6) 456 (63.3) 8 (1.1)	43 (41.3) 58 (55.8) 3 (2.9)
Study period, n (%)				
Preclinical Clinical	118 (40.7) 172 (59.3)	43 (34.4) 82 (65.6)	293 (45.1) 356 (54.9)	91 (82.7) 13 (17.3)
Committed relationship, n (%)				
Yes No	173 (57.7) 127 (42.2)	100 (73.0) 37 (27.0)	346 (48.1) 373 (51.9)	34 (32.7) 70 (67.3)
Housing situation, n (%)				
Alone With others	107 (36.3) 188 (63.7)	23 (16.9) 113 (83.1)	205 (29.3) 494 (70.7)	83 (81.4) 19 (18.6)
Financial situation, n (%)				
No problems Problems	211 (70.1) 90 (29.9)	98 (82.3) 38 (27.7)	460 (64) 259 (36)	85 (81.7) 19 (13.3)
Physical activity, n (%)				
Inactive Active	104 (35) 193 (65)	49 (36) 87 (64)	295 (41.4) 417 (58.6)	52 (51.5) 49 (48.5)

Total N = 1,262.

M, mean value; SD, standard deviation; GER, Germany; AU, Austria; HU, Hungary; JA, Japan.

Descriptive only—no statistical testing was performed.

### Nighttime screen use

Screen time before bedtime (Kolmogorov–Smirnov D = 0.240, Shapiro–Wilk W = 0.779) and time between the end of screen use and bedtime (Kolmogorov–Smirnov D = 0.234, Shapiro–Wilk W = 0.618) deviated significantly from normality (all p <.001, n = 1,262). The median duration of screen use before bedtime was 30 minutes [interquartile Range (IQR) 7.5–52.5 (Germany) and 10–50 (Austria, Hungary, and Japan)] across all locations (no significant difference between the study sites). Device use ended a median of 5 minutes [IQR 0–10 (Japan)] to 10 minutes [IQR 2.5–17.5 (Germany and Austria) and 1.5–18.5 (Hungary)] before bedtime (JA *vs*. GER, AU, and HU, p <.001—see **Table 2**). Across locations, between 96% (Germany) and 98% (Japan) of students used a device with a screen within 1 hour of going to bed (**Table 2**).

**Table 2:** Nighttime screen use among study sites.

Variable/Analysis	GER (n = 301)	AU (n = 137)	HU (n = 720)	JA (n = 104)
Screen time before bedtime in min Median (25Q|75Q) Mean value (SD)	30 (7.5|52.5) 44.8 (61.1)	30 (10|50) 42.8 (33.4)	30 (10|50) 43.8 (40.0)	30 (10|50) 39.2 (36.6)
Pairwise comparison via *Kruskal-Wallis test ^a^*	H(3) = 3.52, p = .318, η^2^ ≤.001			
Germany Austria Hungary Japan	*-* p >.99 p = .68 p >.99	p >.99 - p >.99 p >.99	p = .68 p >.99 - p >.99	p >.99 p >.99 p >.99 -
Time between the end of screen use and bedtime in min Median (25Q|75Q) Mean value (SD)	10.0 (2.5|17.5) 14.1 (15.0)	10.0 (2.5|17.5) 14.8 (16.9)	10.0 (1.5|18.5) 13.4 (14.5)	5.0 (0|10) 8.5 (13.5)
Pairwise comparison via *Kruskal–Wallis test ^a^*	H(3) = 28.34, p <.001, η^2^ = .020			
Germany Austria Hungary Japan	- p >.99 p >.99 p <.001	p >.99 - p >.99 p <.001	p >.99 p >.99 - p <.001	p <.001 p <.001 p <.001 -
Screen use within 1 hour before bed, n (%)	289 (96.0)	133 (97.1)	694 (96.4)	102 (98.1)
*Chi-square test*	*χ*^2^ = (3) = 1.14, p = .767, V = .030			

25Q, 25% quartile; 75Q, 75% quartile; SD, standard deviation; min, minutes; GER, Germany; AU, Austria; HU, Hungary; JA, Japan.

a Bonferroni-corrected significance.

In this case, the most frequently used device was a smartphone/tablet, with usage ranging from 65% in Austria to 89% in Japan. A laptop/PC was used by 1% in Japan to 6% in Hungary, and a TV by 2% in Japan to 12% in Austria. Using multiple devices before bed was reported by 10% in Germany, 17% in Austria, 10% in Hungary, and 8% in Japan (see **Supplementary File 4**).

The proportion of students who never used a screen after waking up at night (for example, to read the news or check social media) ranged from 51% in Austria to 76% in Japan. Whereas 13% in Japan to 25% in Hungary used their electronic device with a screen less than once a week after waking up at night, approximately 12% in Japan to 25% in Austria did so once to several times a week (see **Supplementary File 4**).

The proportion of students who were never disturbed during sleep by calls or notifications ranged from 76% in Hungary to 86% in Japan. Disturbance less than once a week was reported by 12% in Germany to 17% in Hungary, whereas disturbance once or several times a week during their sleep by an electronic device with a screen was reported by 2% in Austria to 7% in Hungary (see **Supplementary File 4**).

### Smartphone addiction symptoms

The internal consistency of the SAS-SV across locations was α ≥.80 (GER = 0.80, AU = 0.83, HU = 0.84, and JA = 0.83). The SAS-SV total score showed a significant deviation from normality (Kolmogorov–Smirnov D = 0.073, p <.001; Shapiro–Wilk W = 0.968, p <.001; n = 1,262). Mean SAS-SV scores were 23.6 (SD 7.4) in Germany, 24.7 (SD 8.4) in Austria, 23.2 (SD 8.5) in Hungary, and 27.0 (SD 8.5) in Japan. While the mean values of the European study sites did not differ significantly from each other, the mean difference between Japan and Germany, as well as Japan and Hungary, was significant at p <.05 (see **Table 3**). The SAS-SV scores of the study sites, depending on sociodemographics, are shown in **Supplementary File 5**.

**Table 3:** Smartphone addiction symptoms across study sites.

Variable/Analysis	GER (n = 301)	AU (n = 137)	HU (n = 720)	JA (n = 104)
Total SAS-SV, M (SD)	23.6 (7.4)	24.7 (8.4)	23.2 (8.5)	27.0 (8.5)
Pairwise comparison via *Kruskal–Wallis test ^a^*	H(3) = 21.71, p <.001, η^2^ = .015			
Germany Austria Hungary Japan	- p >.99 p >.99 p = .004	p = .375 - p = .06 p = .138	p >.99 p = .363 - p <.001	p = .004 p = .138 p <.001 -
Smartphone addiction, n (%)	38 (12.6)	26 (19.0)	113 (15.1)	29 (27.9)
*Chi-square test*	*χ*^2^ = (3) = 14.1, p = .003, V = .106			
Pairwise comparison via Z-test*^a^* Germany Austria Hungary Japan	- p = .66 p >.99 p = .003	p = .66 - p >.99 p = .84	p >.99 p >.99 - p = .02	p = .003 p = .84 p = .02 -

25Q, 25% quartile; 75Q, 75% quartile; SD, standard deviation; min, minutes; GER, Germany; AU, Austria; HU, Hungary; JA, Japan; SAS-SV, Smartphone Addiction Scale–Short Version.

a Bonferroni-corrected significance.

Smartphone addiction was identified in 12.6% of students in Germany, 19.0% of students in Austria, 15.7% of students in Hungary, and 27.9% of students in Japan. The prevalence was significantly higher in Japan than in Germany (p = .003) and in Japan than in Hungary (p = .02) (**Table 3**).

### Sleep

#### PSQI total score

The PSQI total score deviated significantly from a normal distribution (Kolmogorov–Smirnov D = 0.141, p <.001; Shapiro–Wilk W = 0.949, p <.001; n = 1,262). Mean PSQI scores were 5.05 (SD 2.6) in Germany, 4.73 (SD 2.17) in Austria, 5.22 (SD 2.4) in Hungary, and 4.33 (SD 2.0) in Japan, where the mean difference between Hungary and Japan was significant at p <.001 (see **Table 4**). The PSQI scores of the study sites, depending on sociodemographic characteristics, are shown in **Supplementary File 6**.

**Table 4:** PSQI score across the study sites.

Variable/Analysis	GER (n = 301)	AU (n = 137)	HU (n = 720)	JA (n = 104)
Total PSQI, M (SD)	5.12 (2.6)	4.8 (2.2)	5.26 (2.4)	4.33 (2.0)
Pairwise comparison via *Kruskal–Wallis test ^a^*	H(3) = 17.81, p <.001, η^2^ = .0012			
Germany Austria Hungary Japan	- p >.99 p >.99 p = .004	p = .375 - p = .06 p = .138	p >.99 p = .363 - p <.001	p = .004 p = .138 p <.001 -
Poor quality of sleep (PSQI > 5), n (%)	110 (36.5)	46 (33.3)	293 (40.7)	26 (25.0)
*Chi-square test*	*χ*^2^ = (3) = 11.1, p = .011, V = .094			
Pairwise comparison via *Z-test*^a^ Germany Austria Hungary Japan	- p >.99 p >.99 p = .19	p >.99 - p = .71 p = .99	p >.99 p = .71 - p = .01	p = .19 p = .99 p = .01 -

M, mean value; SD, standard deviation; PSQI, Pittsburgh Sleep Quality Index; GER, Germany; AU, Austria; HU, Hungary; JA, Japan.

a Bonferroni-corrected significance.

#### Poor sleep quality

Poor sleep quality (PSQI > 5) was observed in 36.5% of students in Germany, 33.3% in Austria, 40.7% in Hungary, and 25.0% in Japan. The proportion was significantly lower in Japan than in Hungary (p = .01) (see **Table 4**).

### PSQI components

The characteristics of the PSQI components (subjective sleep quality, sleep latency, sleep duration, sleep efficiency, sleep disturbance, use of sleep medication, and daytime dysfunction), depending on study site, are listed in detail in **Supplementary File 7** (note: higher score indicates a poorer outcome).

### Nighttime screen use and sleep

**Figure 1:**
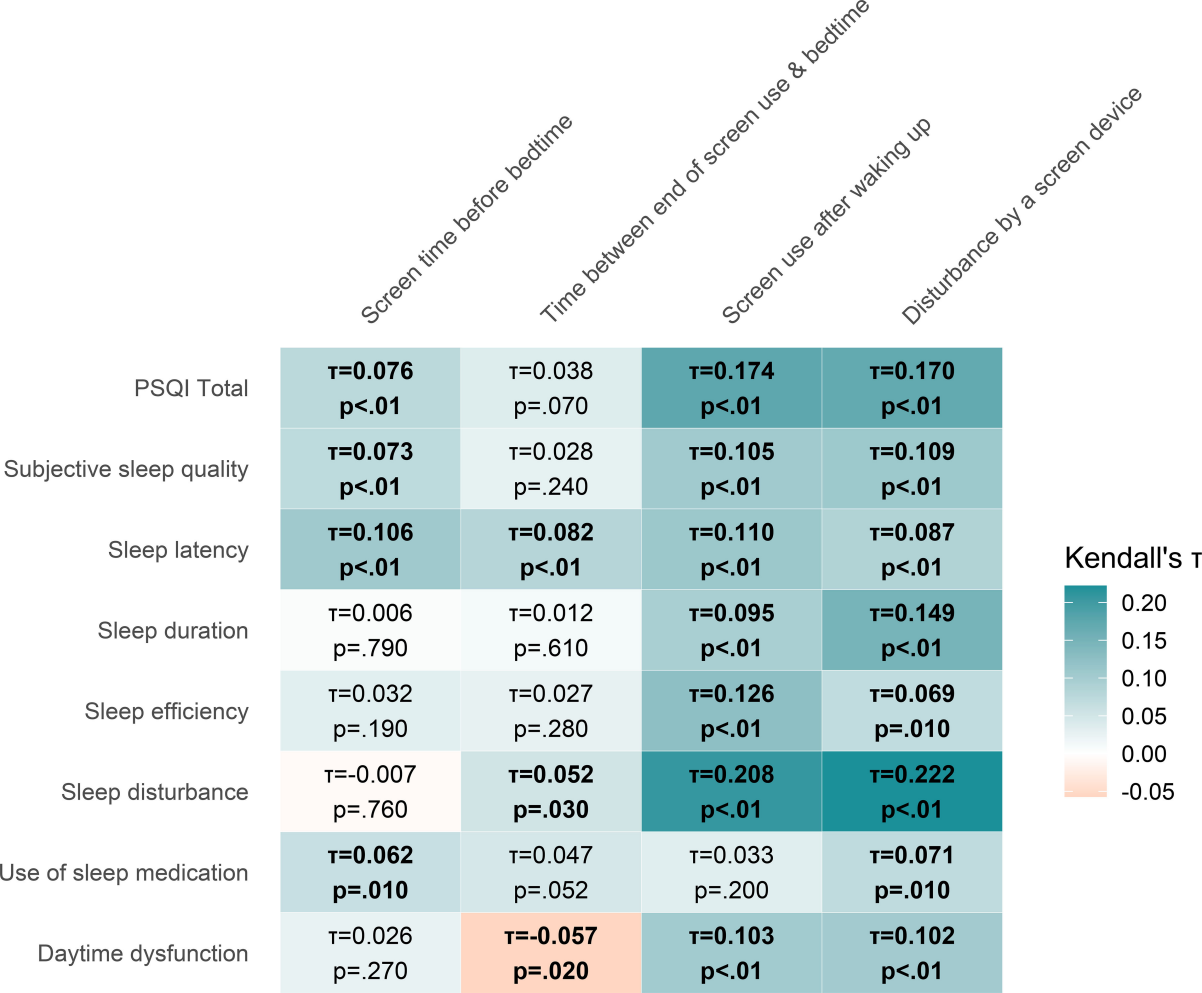
Correlation map—nighttime screen use and sleep.

Given the large sample size, only statistically significant associations with |τ| ≥ 0.10 are highlighted in the text to emphasise effects of at least small practical relevance. An overview of all values is presented in **Figure 1** (across study sites) and site-specific values in **Supplementary File 8**. Across study sites, prolonged screen time before bed was associated with increased sleep latency (τ = 0.106, p <.001). No significant association with |τ| ≥ 0.10 between the time between the end of screen use and bedtime and sleep outcomes was found. Across study sites, using a screen after waking up at night was significantly associated with a poorer PSQI total score (τ = 0.174, p <.001), poorer subjective sleep quality (τ = 0.105, p <.001), increased sleep latency (τ = 0.110, p <.001), reduced sleep efficiency (τ = 0.126, p <.001), increased sleep disturbance (τ = 0.208, p <.001), and increased daytime sleepiness (τ = 0.103, p <.001). The disturbance of sleep by a device with a screen was significantly associated with a poorer PSQI total score (τ = 0.170, p <.001), poorer subjective sleep quality (τ = 0.109, p <.001), increased sleep duration (τ = 0.149, p <.001), greater sleep disturbance (τ = 0.222, p <.001), and greater daytime sleepiness (τ = 0.102, p <.001).

### Nighttime screen use and smartphone addiction symptoms

In the following section, only sitewide values are described. A list of all site-specific values can be found in **Supplementary File 9**. Across study sites, SAS-SV scores were not associated with the duration of screen use before bed (τ = −0.016, p = .45). Increased SAS-SV scores were, however, associated with a shorter distance of ending screen use before bed (τ = −0.150, p <.001), with using a screen after waking up at night (τ = 0.165, p <.001) and with disturbance of sleep by an electronic device with a screen (τ = 0.135, p <.001) (see **Table 5**).

**Table 5:** Correlation matrix of SAS-SV scores and nighttime screen use.

Variable	Screen time before bedtime	Time between the end of screen use and bedtime	Screen use after waking up at night	Disturbance of sleep by an electronic device with a screen
Smartphone addiction symptoms	τ = −0.016 p = .45	**τ = −0.150** **p <.001**	**τ = 0.165** **p <.001**	**τ = 0.135** **p <.001**

Correlation measure: Kendall’s tau-b.

SAS-SV, Smartphone Addiction Scale–Short Version.

Bold:|t| ≥ 0.10.

### Nighttime screen use, sleep quality and smartphone addiction symptoms (adjusted for cofactors)

To assess how the different factors (sociodemographic factors, including study site, nighttime screen use, and smartphone addiction symptoms) are independently associated with sleep quality, an adjusted binary logistic regression model with poor sleep quality (PSQI > 5) as the outcome was conducted. The adjusted GVIF values indicated no relevant multicollinearity (maximum adjusted GVIF = 1.21) and are provided in **Supplementary File 10**. Linearity in the logit for continuous predictors (SAS-SV, age, screen time before bedtime, and time between the end of screen use and bedtime) was tested using the Box–Tidwell approach and showed no evidence of violation (all p >.05).

The amount of screen time before bedtime showed a small but statistically significant association [adjusted odds ratio (aOR) = 1.04, 95% CI 1.01–1.08; p = .015] with poor sleep quality, whereas the time between the end of screen use and bedtime was not independently associated with poor sleep quality (aOR = 1.08, 95% CI 0.98–1.18; p = .112). Nighttime screen use after nocturnal awakenings was associated with poor sleep quality (p <.001), with higher odds for both non-reference categories (aOR = 1.71, 95% CI 1.24–2.34 and aOR = 1.81, 95% CI 1.29–2.54). Device-related sleep disturbance was also independently associated with poor sleep quality (p = .008), with aOR = 1.45 (95% CI 1.02–2.07) and aOR = 2.35 (95% CI 1.25–4.44) for the two non-reference categories, respectively. Smartphone addiction symptoms were not a significant predictor of poor sleep quality (p = .194). Among cofactors, higher age (aOR = 1.06 per year, p = .023), financial problems (aOR = 1.33, p = .042), and single relationship status (aOR = 1.33, p = .038) were associated with poor sleep quality, while a clinical study phase was associated with lower odds (aOR = 0.59, p <.001). Model fit indices were as follows: Hosmer–Lemeshow test p = .587 and Nagelkerke R^2^ = .122.

## Discussion

The aim of the study was to examine the prevalence of nighttime screen use among medical students and to analyse its relationship with sleep and smartphone use. Across study sites, associations were statistically significant but mostly small in magnitude. Pre-bedtime screen time showed a small association with sleep latency (τ = 0.106), whereas screen use after nocturnal awakenings (τ = 0.174) and device-related sleep disturbance (τ = 0.170) showed stronger associations with overall sleep quality (all p <.001). In the adjusted logistic regression model, several nighttime screen use indicators remained independently associated with poor sleep quality, whereas smartphone addiction symptoms (SAS-SV score) showed no independent association.

### Nighttime screen use

Pre-bedtime screen time did not differ significantly between sites (median, 30 minutes), and nearly all students (96%) put their devices aside only 5–10 minutes before bedtime, thereby not meeting the recommendation of the National Sleep Foundation (14). Psychophysiological arousal and suppression of melatonin vary with the device [distance to the screen (i.e., short distance (smartphone) *vs*. longer distance (TV)] and the background of use [active usage (i.e., writing and gaming) *vs*. passive usage (i.e., watching)] (41). The majority of students—65% to 89%—used a smartphone or tablet, devices typically held at close range and associated with poorer sleep outcomes (42). Detailed usage context (e.g., active *vs*. passive screen time) was not assessed, as pre-tests showed that it could not be captured reliably with a questionnaire. Nevertheless, while avoiding screen use entirely 1 hour before bedtime would be ideal for medical students, the passive (rather than active) use of a distant screen—typically a television instead of a smartphone or tablet—may be preferable for promoting sleep hygiene among medical students.

### Smartphone addiction symptoms

Compared with a meta-analysis from 2022 (21) that reported a prevalence of smartphone addiction of approximately 42% among Asian medical students and with European data from Serbia (22%) and France (29%) (43, 44), the rates in our sample were lower overall. Consistent with the literature, the Asian site in our study (Japan, 27.9%) still showed higher smartphone addiction symptoms than the European sites (Germany, 12.6%; Austria, 19.0%; and Hungary, 15.7%). Nevertheless, our findings reveal a possible disconnect: although only 12.6% to 27.9% of students met the SAS-SV cut-off for “smartphone addiction”, 96% reported using their device right up to bedtime. This discrepancy may partly reflect that the SAS-SV captures primarily addiction-like symptoms (e.g., impaired control and compulsive use) and does not comprehensively assess broader patterns of smartphone-related problems, such as habitual overuse or context-specific behaviours. Consistent with this interpretation, SAS-SV scores were not an independent predictor of poor sleep quality in the logistic regression model, whereas nighttime screen use remained significantly associated with poor sleep quality. Nevertheless, the side effects associated with high smartphone use, such as communication problems (45) or deficits in emotion regulation (46), which are particularly important for the medical profession, should be considered. Recent work among healthcare professionals links excessive smartphone use to reduced empathy (47) and to heightened alexithymia (reduced ability to recognise emotion in others) (48), which may directly compromise doctor–patient interactions.

### Sleep

The sleep quality (PSQI total score) of medical students in Japan was better [mean 4.33 (SD 2.0)] than that of medical students in Europe, particularly in Hungary [mean 5.26 (SD 2.4)]. Poor sleep quality (PSQI > 5) was observed in approximately 41% of students in Hungary, 37% in Germany, 33% in Austria, and 25% in Japan. In contrast, current international meta-analyses (8, 49) indicate a global prevalence of 53%–56% of poor sleep quality (PSQI > 5) among medical students. In the adjusted logistic regression model, poor sleep quality was independently associated with higher age, female gender, preclinical study phase, and financial problems (**Table 6**), suggesting that differences in sample composition between sites may partly contribute to observed site differences in sleep outcomes. Further contextual factors that could explain the wide variance in effect sizes may be survey timing (e.g., during exam periods; 50), living arrangements (sleeping alone *vs*. in dormitories; 51), and cultural aspects such as daily routines, attitudes towards sleep, and social norms (52). For example, Japanese students were the sub-sample of the present study, who had both above-average SAS-SV scores and better sleep hygiene on average. One possible explanation for this pattern is that higher SAS-SV scores do not necessarily reflect the specific nighttime behaviours that are more relevant for sleep. In our data, Japan showed higher SAS-SV scores but less frequent nocturnal device use, whereas these nighttime behaviours (rather than SAS-SV scores) were significant predictors of sleep quality in the adjusted model. Cross-cultural differences in sleep norms and symptom reporting may also contribute, as Japanese students had the best average PSQI scores, yet they rated their subjective sleep quality the worst [mean 1.17 (SD 0.6)]. Their only objectively poorer outcome was sleep duration: they slept the least [mean 6.90 hours (SD 1.0)], and approximately 40% failed to meet the 7 hours of sleep recommended by the American Academy of Sleep Medicine (7). This aligns with earlier findings (53) that Japanese students sleep significantly less than Canadian peers while reporting less daytime fatigue—a pattern we likewise observed in our cohort, where Japanese students reported the least daytime tiredness. Beyond subjective perceptions, good and sufficient sleep among medical students is essential for academic performance (5, 6), their own health (7), and the health of their future patients (26, 27). The available evidence indicates the need for interventions to improve sleep quality and sleep hygiene among medical students. Interventions to improve sleep hygiene may therefore be warranted; however, translating sleep hygiene knowledge into practice remains challenging (54).

**Table 6:** Multivariable logistic regression predicting poor sleep quality (PSQI > 5).

Predictor	aOR	95% CI	p
**Screen use after waking up at night**			<.001
<1/week (*vs*. never)	1.706	1.243–2.340	<.001
≥1/week (*vs*. never)	1.805	1.286–2.535	<.001
**Sleep disturbed by device**			.008
<1/week (*vs*. never)	1.450	1.017–2.067	.040
≥1/week (*vs*. never)	2.351	1.246–4.436	.008
**Smartphone addiction symptoms**	1.093	0.956–1.251	.194
**Clinical study phase** (*vs*. preclinical study phase)	0.585	0.434–0.790	<.001
**Study site**			.031
AU (*vs*. GER)	0.990	0.615–1.593	.967
HU (*vs*. GER)	1.272	0.923–1.752	.141
JA (*vs*. GER)	0.600	0.336–1.069	.083
**Age** (per year)	1.055	1.008–1.105	.023
**Gender**			.094
Female (*vs*. male)	1.362	1.030–1.801	.030
Diverse/unknown (*vs*. male)	1.128	0.305–4.170	.857
**No committed relationship** (*vs*. committed relationship)	1.326	1.015–1.732	.038
**Physically active** (*vs*. inactive)	1.056	0.812–1.374	.685
**Financial problems** (*vs*. no problems)	1.334	1.010–1.762	.042
**Living alone** (*vs*. with others)	0.773	0.581–1.028	.077
**Screen time before bedtime** (per 10 min)	1.078	0.983–1.182	.112
**Time between end of screen use and bedtime** (per 10 min)	1.041	1.008–1.075	.015

Model fit: Hosmer–Lemeshow test p = .587, Nagelkerke R^2^ = .122.

a OR, adjusted odds ratio; PSQI, Pittsburgh Sleep Quality Index; AU, Austria; GER, Germany; HU, Hungary; JA, Japan.

### Nighttime screen usage, smartphone addiction symptoms, and sleep

The length of screen use before bed was significantly associated with sleep quality across study sites, but with a small effect size (τ = 0.076, p <.001). Most of the observed associations stem from an increased sleep latency (τ = 0.106, p <.001), which could reflect an increased psychophysiological arousal or a reduced release of melatonin according to the model of Cain and Gradisar (13). Nevertheless, sleep quality is multifactorial; the present analysis captured only part of the picture. Variables such as caffeine or alcohol intake, chronotype, ambient light, noise, or stress (55–58) can each exert effects comparable to—or greater than—screen exposure before bed. Large studies that examine pre-bedtime screen use alongside other sleep determinants are needed to quantify its unique predictive power in terms of sleep quality.

The time between the end of screen use and bedtime was not significantly related to sleep quality across all locations (τ = 0.038, p = .07). In addition to the possibility that there is no real effect, the low, non-significant correlation could be explained by the low interindividual variance (=variance restriction), as almost all students used a screen within 1 hour before going to bed. A recent National Sleep Foundation analysis (59) showed that using a smartphone 1 hour before bedtime was associated with worsened sleep quality compared with no pre-bedtime smartphone use [t(1,007) = −2.19, p = .029]. A large population study (60) of n = 122,058 adults supported this pattern: daily pre-bedtime screen use was linked to a 33% higher prevalence of poor sleep quality (prevalence ratio 1.33, 95% CI 1.27–1.39) relative to no screen use. This may suggest that screen use within 1 to 2 hours before bedtime is more strongly associated with sleep quality than the exact length of time between last screen use and lights out, especially if the time interval is very short.

In the European sites (GER, AU, and HU), about one in four students used a screen at least weekly after nocturnal awakenings. Across all locations, this behaviour correlated with poorer global sleep quality (τ = 0.174, p <.001) and with longer sleep latency, more disturbances, lower efficiency, worse subjective quality, and greater daytime sleepiness (all τ > 0.10, p <.001). It was also more common among students with higher SAS-SV scores (τ = 0.165, p <.001) across European study sites. Beyond its association with poorer sleep, nocturnal screen use has been linked in adolescent and student populations to higher stress levels (61), poorer cognitive performance (62), more severe depressive symptoms (63, 64), and increased suicidal ideation (65), while some of these effects are mediated by sleep disruption (66).

However, device-related sleep disturbance was infrequently reported: only in Hungary, twice as many students as at the other study sites (HU: 6.7% *vs*. GER, AU, and JA: between 1.5% and 3.3%) reported that they were being weekly disturbed by a device with a screen. Across all locations, this form of sleep disturbance was associated with poorer overall sleep quality (τ = 0.170, p <.001), poorer outcomes in four of seven PSQI subscales (each τ > 0.1, p <.001), and higher SAS-SV scores (τ = 0.135, p <.001). Sleep interruption can disrupt deep and REM sleep stages, which impairs recovery and memory formation (24). In the long term, they can increase the risk of depression, anxiety disorders, and irritability as well as substance abuse, such as alcohol and nicotine abuse (25).

In comparison, roughly 20% of U.S. adolescents and young adults reported sleep disruptions caused by electronic devices “a few nights per week” (67), 35% of U.S. college and high-school students said they were “occasionally to frequently” awakened by—or actively checked—a device during the night (68), and 59% of Moroccan medical students admitted interrupting their sleep to read messages (69). Due to the already slightly older publication dates (between 2013 and 2019), and the items in the present and referenced studies cover different time frames and partly different content, these figures are comparable only to a limited extent.

### Limitations

The items regarding nighttime screen use covered the average daily use of a screen, with no differentiation made between working week and weekend, as well as between different usage backgrounds. Furthermore, the items were self-developed and, beyond pre-testing, not formally validated (e.g., test–retest reliability or measurement invariance across languages). This may have introduced measurement uncertainty and thus affected the generalisability and strength of the observed findings.

There is some evidence that self-reports on the amount of screen time deviate from objective measurement methods (70). In addition, our survey data is subject to recall bias, which may distort the exact timing and duration of screen use and sleep, and social desirability bias, which can lead students to under-report behaviours perceived as unhealthy (e.g., late-night scrolling) and over-report desirable habits (e.g., sleep duration).

Our overall response was modest (13%–37% across sites), and a part of the received questionnaires were excluded because of incomplete items. Such rates are typical for voluntary, unsponsored online surveys among medical students, yet they limit representativeness. Students who were especially health-conscious (or those struggling with sleep) may have been more (or less) likely to participate/finish the survey. Non-response and attrition could therefore shift effects in either direction. Because analyses were based on complete cases, some additional selection bias cannot be excluded, but differences between included and excluded respondents were small overall (**Supplementary File 3**).

Sample sizes differed substantially between study sites (HU n = 720 *vs*. JA n = 104), which may have affected the precision of site-specific estimates and may have influenced pooled estimates due to the larger contribution of some sites. To address this, we used robust/non-parametric procedures where appropriate, applied Bonferroni correction for pairwise site comparisons, and included study site as a covariate in the adjusted regression analyses. The Bonferroni correction applied in this study represents a conservative measure to control the alpha error. While some (non-significant) group differences would have been significant if less stringent correction methods had been used, it can also be assumed that certain effect sizes reached the significance level of p <.05 due to the large number of cases. To counteract the potential overestimation of such effects, significant results with effect sizes of τ < 0.1 were categorised as less important.

Data collection periods differed between sites and were largely site-specific, so potential seasonal effects could not be separated from study site effects. Due to the cross-sectional design, the findings are correlational and do not allow conclusions about causality between nighttime screen use and sleep quality. Based on the results and existing literature (71–73), it can be assumed that nighttime screen use is associated with sleep quality, but that the influence is low to moderate in view of the numerous other factors that determine sleep quality.

## Conclusions and transfer

Nighttime screen use among medical students was associated with poorer sleep quality, with mostly small-to-moderate effect sizes. The smartphone/tablet was the most frequently used device across countries. However, associations between smartphone addiction symptoms and nighttime screen use indicators were not consistent across sites and appeared more pronounced in the European samples. Although prevalence and exact PSQI scores varied between Germany, Austria, Hungary, and Japan, the overall pattern was similar across sites, which makes the findings relevant to other medical schools.

Potential risk reduction of nighttime screen use among medical students may be supported through a mix of behavioural and structural measures. Faculties could raise awareness—among both students and teaching staff—about the interaction of screen use and sleep. Behavioural interventions that have been shown to reduce late-evening screen time and improve sleep and mood, such as a sunrise alarm clock programme (sunrise alarm plus removal of devices from the bedroom) (2), could be embedded in the medical curriculum on an opt-in basis. Such measures may benefit medical students directly and, more broadly, may strengthen their credibility when counselling patients. Better sleep and reduced smartphone use may enhance psychological well-being (7, 25, 66), communication, and empathy (45–47), thereby helping future physicians to act as more authentic and convincing role models (26, 27).

## Data Availability

The datasets presented in this article are not readily available. To ensure compliance with the data protection plan outlined in the ethics application, all individuals requiring access to the data must first be reported to the Ethics Committee of TU Dresden (No. EK 15012014). Upon notification and signing a data protection agreement, access to the data will be granted based on justified requests. Requests to access the datasets should be directed to the corresponding author.
