# Supplementary material for: Nighttime screen use, sleep quality, and smartphone addiction symptoms among medical students: an international cross-sectional study
Source: Front Psychiatry. 2026 Feb 6;17:1735186. doi: 10.3389/fpsyt.2026.1735186 (PMC12920586; doi:10.3389/fpsyt.2026.1735186)
Supplement: Supplementary file 1 [file Supplementaryfile1.docx]

Supplementary 1: CHERRIES Checklist – Reporting Results of Internet E‑Surveys

| Item category | Checklist item | Explanation (as applied in this study) | Location in manuscript |
| --- | --- | --- | --- |
| Design | Describe survey design | Cross-sectional, international multicentre web survey among enrolled medical students; conducted in 2024 at four sites (Dresden/GER, Linz/AUT, Pécs/HU, Matsumoto/JA); available in German, Hungarian, Japanese, and English | Methods: Survey Design |
| IRB approval & Informed consent process | IRB approval | Ethics approvals obtained at all sites: Dresden (EK15012014), Linz (EK1166/2023), Pécs (BMEÜ/2448-1/2022/EKU), Matsumoto (6176) | Methods: Ethics and Informed Consent |
|  | Informed consent | Participants read an online information sheet (purpose, voluntariness, data handling, risks/benefits, contacts) and provided consent by ticking an “I agree” checkbox; withdrawal possible via email using an optional pseudonymisation code | Methods: Ethics and Informed Consent |
|  | Data protection | Survey responses stored on an encrypted university server in Germany; GDPR compliant; no direct identifiers collected; LimeSurvey stored a truncated (anonymised) IP address and session cookies to resume sessions and prevent multiple submissions | Methods: Ethics and Informed Consent / Survey administration |
| Development and Pre-testing | Development and testing | Instrument combined validated scales (PSQI; SAS‑SV translation) with five self-developed nighttime screen-use items. Think‑aloud pre-test (n=15) and iterative refinement; forward–backward translation; expert panel review; cognitive debriefing (≥5 per language); beta-testing across languages | Methods: Development, measuring instruments and pre-testing |
| Recruitment process & description of the sample having access to the questionnaire | Open survey versus closed survey | Closed survey: link distributed only to enrolled medical students via institutional channels; not publicly advertised | Methods: Recruitment and eligibility |
|  | Contact mode | Initial contact primarily online (faculty e‑mail newsletters, student social‑media groups) and in-person announcements during lectures/seminars; survey completion online | Methods: Recruitment and eligibility |
|  | Advertising the survey | Distributed via faculty newsletters, student-run social-media groups, and brief announcements in teaching events; no banner ads or paid media; no incentives offered | Methods: Recruitment and eligibility |
| Survey administration | Web/E‑mail | Web-based survey implemented in LimeSurvey v2.50+; responses captured automatically by the platform. | Methods: Survey administration |
|  | Context | Survey accessed through direct link shared by faculties/student groups | Methods: Recruitment and eligibility / Survey administration |
|  | Mandatory/voluntary | Participation was voluntary | Methods: Survey administration |
|  | Incentives | No incentives were offered; non‑participation carried no penalties | Methods: Ethics and Informed Consent / Recruitment |
|  | Time/Date | Site-specific field periods: Pécs (HU) Feb–May 2024; Linz (AT) Mar–Jul 2024; Dresden (DE) Apr–Jul 2024; Matsumoto (JA) Jun–Jul 2024 | Methods: Survey administration |
|  | Randomization of items or questionnaires | No randomization of items or questionnaires was applied | Methods: Survey administration |
|  | Adaptive questioning | Partly conditional branching were applied: follow-up questions were displayed only if a preceding response made them relevant | Methods: Survey administration |
|  | Number of items | Varied between participants due to branching; average completion time ~15 minutes (from beta tests) | Methods: Development… / Survey administration |
|  | Number of screens (pages) | Varied between participants due to branching; pages/items per page differed by path | Methods: Survey administration |
|  | Completeness check | No completeness checks were enforced by the software; participants could skip items | Methods: Survey administration |
|  | Review step | Respondents could navigate backwards at any time to revise earlier answers | Methods: Survey administration |
| Response rates | Unique site visitor | Not applicable, as no access code or central landing page was used |  |
|  | View rate | Not applicable as survey links were distributed via multiple decentralized channels and website traffic data could not be restricted to genuine student visitors (risk of bot traffic) |  |
|  | Participation rate | Not applicable as survey links were distributed via multiple decentralized channels and website traffic data could not be restricted to genuine student visitors (risk of bot traffic) |  |
|  | Completion rate | Not calculated, as only complete cases were analysed | Methods: Statistical analyses |
| Preventing multiple entries from the same individual | Cookies used | LimeSurvey stored a session cookie to prevent multiple submissions from the same browser and to allow resuming interrupted sessions | Methods: Ethics and Informed Consent / Survey administration |
|  | IP check | A truncated (anonymised) IP address was stored to help identify repeat entries without collecting full identifiers | Methods: Ethics and Informed Consent |
|  | Log file analysis | No additional post-hoc log file analysis was performed |  |
|  | Registration | No user registration or login; participation was pseudonymous; optional pseudonymisation code displayed at debriefing | Methods: Ethics and Informed Consent |
| Analysis | Handling of incomplete questionnaires | Complete-case approach: only records with complete information on nighttime screen use, PSQI, and SAS‑SV were analyzed | Methods: Statistical analyses |
|  | Questionnaires submitted with an atypical timestamp | No analysis of atypical timestamps was performed | Methods: Statistical analyses |
|  | Statistical correction | No weighting or propensity score adjustment applied; analyses were exploratory and bivariate (with multiple-comparison control via Bonferroni for pairwise comparisons) | Methods: Statistical analyses |
