# Supplementary material for: Nighttime screen use, sleep quality, and smartphone addiction symptoms among medical students: an international cross-sectional study
Source: Front Psychiatry. 2026 Feb 6;17:1735186. doi: 10.3389/fpsyt.2026.1735186 (PMC12920586; doi:10.3389/fpsyt.2026.1735186)
Supplement: Supplementary file 2 [file Supplementaryfile2.docx]

Supplementary 2: English items: Nighttime screen use (2.1), PSQI (2.2), SAS-SV (translated 2.3)

2.1

*How long do you usually use an electronic device with a screen (cell phone, tablet, laptop, TV) before going to bed?*

___ minutes

*How many minutes before going to bed do you usually stop using an electronic device with a screen (such as cell phone, tablet, laptop, TV)?*

___ minutes

*How long do you usually use an electronic device with a screen (cell phone, tablet, laptop, TV) before going to bed?*

___ minutes

*Which electronic device with a screen do you usually use before going to bed? (Single choice)*

- Cell phone/tablet
- Laptop/PC
- TV
- Several of the above devices

*How often do you use an electronic device with a screen after waking up at night, for example to read messages or check social media?*

- Never
- Less than once a week
- 1-2x/week
- 3-4x/week
- 5-7x/week

*How often is your sleep interrupted by calls or notifications from an electronic device with a screen? (excluding alarm clock function)*

- Never
- Less than once a week
- 1-2x/week
- 3-4x/week
- 5-7x/week

2.2

*The following questions relate to your usual sleep habits during the past month only. Your answers should indicate the most accurate reply for the majority of days and nights in the past month. Please answer all questions.*

*During the past month, when have you usually gone to bed at night? (e.g. 18:50)*

----- o’clock

*During the past month, how long (in minutes) has it usually take you to fall asleep each night?*

---- minutes

*During the past month, when have you usually gotten up in the morning? (e.g. 07:30)*

----- o’clock

*During the past month, how many hours of actual sleep did you get at night? (This may be different than the number of hours you spend in bed.)*

---- hours

*During the past month, how often have you had trouble sleeping because you…*

|  | Not during the past month | Less than once a week | Once or twice a week | Three or more times a week |
| --- | --- | --- | --- | --- |
| Cannot get to sleep within 30 minutes |  |  |  |  |
| Wake up in the  middle of the night or early morning |  |  |  |  |
| Have to get up to use the bathroom |  |  |  |  |
| Cannot breathe comfortably |  |  |  |  |
| Cough or snore loudly? |  |  |  |  |
| Feel too cold |  |  |  |  |
| Feel too hot |  |  |  |  |
| Have bad dreams |  |  |  |  |
| Have pain |  |  |  |  |
| Other reasons (please describe) |  |  |  |  |

*During the past month, how would you rate your sleep quality overall?*

- very good
- fairly good
- fairly bad
- Very bad

*During the past month, how often have you taken medicine to help you sleep (prescribed or*

*“over the counter”)?*

- Not during the past month
- Less than Once a week
- Once or twice a week
- Three or more times a week

*During the past month, how often have you had trouble staying awake while driving, eating meals or engaging in social activity?*

- Not during the past month
- Less than Once a week
- Once or twice a week
- Three or more times a week

*During the past month, how much of a problem has it been for you to keep up enough enthusiasm to get things done?*

- No problem at all
- Only a very slight problem
- Somewhat of a problem
- A very big problem

2.3

*Based on your current situation to what extent do you agree with the following statements?*

1= Strongly disagree

6= Strongly agree

1. Missing planned work due to smartphone use
2. Having a hard time concentrating in class, while doing assignments, or while working due to smartphone use
3. Feeling pain in the wrists or at the back of the neck while using a smartphone
4. Won’t be able to stand not having a smartphone
5. Feeling impatient and fretful when I am not holding my smartphone
6. Having my smartphone in my mind even when I am not using it
7. I will never give up using my smartphone even when my daily life is already greatly affected by it
8. Constantly checking my smartphone so as not to miss any news or messages
9. Using my smartphone longer than I had intended
10. The people around me tell me that I use my smartphone too much
