# Supplementary material for: Nighttime screen use, sleep quality, and smartphone addiction symptoms among medical students: an international cross-sectional study
Source: Front Psychiatry. 2026 Feb 6;17:1735186. doi: 10.3389/fpsyt.2026.1735186 (PMC12920586; doi:10.3389/fpsyt.2026.1735186)
Supplement: Supplementary file 3 [file Supplementaryfile3.docx]

**Supplementary 3 Comparison of included (complete-case) and excluded respondents**

| Variable | Included n (% within category) / M (SD) | Excluded n (% within category) / M (SD) | Total n | Test statistic | p | Effect size |
| --- | --- | --- | --- | --- | --- | --- |
| Study site |  |  |  | χ²(38)=39.24 | <.001 | Cramer's V=.145 |
| Germany | 301 (62.4) | 181 (37.6) | 481 |  |  |  |
| Austria | 137 (68.8) | 62 (31.2) | 199 |  |  |  |
| Hungary | 720 (73.3) | 262 (26.7) | 989 |  |  |  |
| Japan | 104 (52.3) | 95(47.7) | 199 |  |  |  |
| Age, M (SD) | 22.7 (3.4) | 23.3 (3.6) | 1868 | t(1095.88)=-3.359 | <0.001 | d=-.172 |
| Gender |  |  |  | χ²(2)=9.37 | .009 | Cramer's V=.071 |
| Male | 425 (64.2) | 237 (35.8) | 662 |  |  |  |
| Female | 824 (69.4) | 363 (30.6) | 1187 |  |  |  |
| Diverse / unknown | 13 (92.9) | 1 (7.1) | 14 |  |  |  |
| Study phase |  |  |  | χ²(1)=2.59 | .107 | Cramer's V=.039 |
| Preclinical | 545 (66.0) | 281 (34.0) | 826 |  |  |  |
| Clinical | 623 (69.6) | 272(30.4) | 895 |  |  |  |
| Housing situation |  |  |  | χ²(1)=27.2 | <.001 | Cramer's V=.122 |
| Living alone | 418 (60.3) | 275(39.7) | 693 |  |  |  |
| Not living alone | 814 (72.1) | 315 (27.9) | 1129 |  |  |  |
| Financial situation |  |  |  | χ²(1)=0.275 | .600 | Cramer's V=.012 |
| No problems | 461 (66.9) | 228 (33.1) | 689 |  |  |  |
| Problems | 800 (68.1) | 375 (31.9) | 1175 |  |  |  |
| Physical activity |  |  |  | χ²(1)=2.6 | .103 | Cramer's V=.041 |
| Inactive | 508 (77.9) | 144 (22.1) | 652 |  |  |  |
| Active | 746 (81.3) | 172 (18.7) | 918 |  |  |  |

Percentages are within category ; n (%) indicates the proportion included and excluded among respondents from the same category. Percentages are shown for categorical variables and continuous variables are reported as mean (SD). *Testing; categorical variables - Pearson χ² tests with Cramer's V ; continous variables - Welch’s t-test with Cohen's d*
