# Supplementary material for: Nighttime screen use, sleep quality, and smartphone addiction symptoms among medical students: an international cross-sectional study
Source: Front Psychiatry. 2026 Feb 6;17:1735186. doi: 10.3389/fpsyt.2026.1735186 (PMC12920586; doi:10.3389/fpsyt.2026.1735186)
Supplement: Supplementary file 4 [file Supplementaryfile4.docx]

Supplementary 4:

3.1 Use of electronic devices with screen (type) by study site

|  | GER  (n=301) | AU  (n=137) | HU  (n=720) | JA  (n=104) |
| --- | --- | --- | --- | --- |
| Cell phone/tablet, n (%) | 244 (81.1) | 89 (65.0) | 588 (81.7) | 93 (89.4) |
| Laptop/PC, n (%) | 12 (4.0) | 8 (5.8) | 45 (6.3) | 1 (1.0) |
| TV, n (%) | 14 (4.7) | 17 (12.4) | 16 (2.2) | 2 (1.9) |
| Several of the above devices, n (%) | 31 (10.3) | 23 (16.8) | 71 (9.9) | 8 (7.7) |
| descriptive only – no statistical testing performed | | | | |

3.2 Using an electronic device with a screen after waking up at night

|  | GER  (n=301) | AU  (n=137) | HU  (n=720) | JA  (n=104) |
| --- | --- | --- | --- | --- |
| Never, n (%) | 166 (55.1) | 70 (51.1) | 374 (51.9) | 79 (76.0) |
| Less than once a week, n (%) | 64 (21.3) | 33 (24.1) | 177 (24.6) | 13 (12.5) |
| Once to several times a week, n (%) | 71 (23.6) | 34 (24.8) | 169 (23.5) | 12 (11.5) |
| descriptive only – no statistical testing performed | | | | |

3.3 Interruption of sleep by an electronic device with a screen

|  | GER  (n=301) | AU  (n=137) | HU  (n=720) | JA  (n=104) |
| --- | --- | --- | --- | --- |
| Never, n (%) | 256 (85.0) | 113 (82.5) | 547 (76.0) | 89 (85.6) |
| Less than once a week, n (%) | 35 (11.6) | 22 (16.1) | 125 (17.4) | 13 (12.5) |
| Once to several times a week, n (%) | 10 (3.3) | 2 (1.5) | 48 (6.7) | 2 (1.9) |
| descriptive only – no statistical testing performed | | | | |
