# Supplementary material for: Nighttime screen use, sleep quality, and smartphone addiction symptoms among medical students: an international cross-sectional study
Source: Front Psychiatry. 2026 Feb 6;17:1735186. doi: 10.3389/fpsyt.2026.1735186 (PMC12920586; doi:10.3389/fpsyt.2026.1735186)
Supplement: Supplementary file 5 [file Supplementaryfile5.docx]

Supplementary 5: Problematic smartphone use of the study sites depending on socio-demographics

|  | GER  (n=301) | AU  (n=137) | HU  (n=720) | JA  (n=104) |
| --- | --- | --- | --- | --- |
| Gender, M (SD)  Male  Female  Not specified, diverse  *Welch’s t-test* ^b^ | 21.9 (7.0)  24.3 (7.5)  22.0 (1.4)  p=.01 | 24.1 (8.5)  25.0 (8.4)  -  *p=.*57 | 22.6 (8.6)  23.6 (8.4)  24.8 (7.9)  *p=.*14 | 25.9 (8.9)  27.8 (8.1)  27.3 (11.1)  *p=.*27 |
| Study period, M (SD)  Preclinical  Clinical  *Welch’s t-test* | 23.6 (7.5)  23.7 (7.4)  *p=.*83 | 24.4 (7.3)  24.4 (8.5)  *p=.*97 | 23.9 (8.4)  22.9 (8.7)  *p=.*13 | 27.4 (8.4)  23.9 (9.0)  *p=.*20 |
| Fixed partnership, M (SD)  Yes  No  *Welch’s t-test* | 23.4 (7.4)  23.9 (7.6)  *p=.*52 | 24.5 (8.8)  25.4 (7.2)  *p=.*55 | 23.4 (8.4)  23.0 (8.7)  *p=.*48 | 26.9 (7.9)  27.1 (8.8)  *p=*.90 |
| Housing situation, M (SD)  Alone  With others  *Welch’s t-test* | 23.0 (7.0)  24.1 (7.7)  *p=.*22 | 22.5 (7.9)  25.1 (8.5)  *p=.17* | 23.5 (8.7)  23.2 (8.5)  *p=.*61 | 27.0 (8.6)  26.6 (8.6)  *p=.*82 |
| Financial situation, M (SD)  No problems  Problems  *Welch’s t-test* | 23.6 (7.6)  23.6 (7.0)  *p=.*99 | 25.1 (8.6)  23.6 (7.8)  *p=.*32 | 23.2 (8.7)  23.3 (8.3)  *p=.*93 | 27.0 (8.4)  27.1 (9.4)  *p=.*98 |
| Physical activity, M (SD)  Inactive  Active  *Welch’s t-test* | 24.4 (7.6)  23.2 (7.3)  *p=.*20 | 25.2 (9.0)  24.3 (8.0)  *p=.*56 | 24.0 (8.6)  22.7 (8.5)  *p=.*046 | 26.9 (8.3)  26.9 (8.7)  *p=.*97 |
| M: mean value, SD: standard deviation ^b^: Test between male/female | | | | |
