# Supplementary material for: Nighttime screen use, sleep quality, and smartphone addiction symptoms among medical students: an international cross-sectional study
Source: Front Psychiatry. 2026 Feb 6;17:1735186. doi: 10.3389/fpsyt.2026.1735186 (PMC12920586; doi:10.3389/fpsyt.2026.1735186)
Supplement: Supplementary file 6 [file Supplementaryfile6.docx]

Supplementary 6: PSQI scores of the study sites depending on socio-demographics

|  | GER  (n=301) | AU  (n=137) | HU  (n=720) | JA  (n=104) |
| --- | --- | --- | --- | --- |
| Gender, M (SD)  Male  Female  Not specified, diverse  *Welch’s t-test* ^b^ | 4.7 (2.8)  5.3 (2.5)  2.0 (1.4)  *p=.*07 | 4.8 (2.7)  4.7 (1.8)  -  *p=.*92 | 5.1 (2.5)  5.4 (2.3)  4.1 (4.1)  *p=.*92 | 3.9 (1.6)  4.5 (2.2)  6.3 (2.1)  *p=.*20 |
| Study period, M (SD)  Preclinical  Clinical  *Welch’s t-test* | 5.5 (3.0)  4.8 (2.3)  *p=.*07 | 4.9 (2.6)  4.6 (1.9)  *p=.*60 | 5.7 (2.4)  5.0 (2.4)  *p<*.001 | 4.3 (2.1)  4.5 (1.7)  *p=.*67 |
| Comitted relationship, M (SD)  Yes  No  *Welch’s t-test* | 5.1 (2.6)  5.0 (2.7)  *p=.*83 | 4.7 (2.1)  4.6 (2.5)  *p=.*84 | 5.0 (2.3)  5.5 (2.4)  *p=.*009 | 4.3 (2.3)  4.3 (1.9)  *p=.*96 |
| Housing situation, M (SD)  Alone  With others  *Welch’s t-test* | 5.1 (2.6)  4.9 (2.6)  *p=.*464 | 4.9 (2.2)  4.7 (2.2)  *p=.*711 | 5.5 (2.4)  5.1 (2.4)  *p=.*03 | 4.2 (1.8)  4.8 (2.7)  *p=.*42 |
| Financial situation, M (SD)  No problems  Problems  *Welch’s t-test* | 5.0 (2.5)  5.4 (2.8)  *p=.*06 | 4.5 (2.0)  5.5 (2.4)  *p=.*02 | 5.2 (2.3)  5.6 (2.4)  *p=.*01 | 4.4 (2.0)  4.1 (2.1)  *p=.*50 |
| Physical activity, M (SD)  Inactive  Active  *Welch’s t-test* | 5.1 (2.6)  5.1 (2.6)  *p=.*65 | 4.8 (1.9)  4.7 (2.3)  *p=.*45 | 5.4 (2.5)  5.2 (2.3)  *p=.*38 | 4.2 (2.1)  4.4 (1.9)  *p=.*62 |
| M: mean, SD: standard deviation, ^b^: test between male/female | | | | |
