# Supplementary material for: Nighttime screen use, sleep quality, and smartphone addiction symptoms among medical students: an international cross-sectional study
Source: Front Psychiatry. 2026 Feb 6;17:1735186. doi: 10.3389/fpsyt.2026.1735186 (PMC12920586; doi:10.3389/fpsyt.2026.1735186)
Supplement: Supplementary file 7 [file Supplementaryfile7.docx]

| Supplementary 7: PSQI components across the study sites | | | | |
| --- | --- | --- | --- | --- |
|  | | | | |
|  | GER  (n=301) | AU  (n=137) | HU  (n=720) | JA  (n=104) |
| Score subjective  Sleep quality (0-4) \| M (SD)  Pairwise comparison via  *Kruskall Wallis test* ^a^  Dresden  Austria  Hungary  Japan | 1.04 (0.6)  -  *p>*.99  *p=.*64  *p=.*56 | 1.00 (0.6)  *p>*.99  -  *p=.*04  *p=.*07 | 1.12 (0.6)  *p>*.99  *p=.*04  -  *p>*.99 | 1.17 (0.6)  *p=.*56  *p=.*07  *p>*.99  - |
| Subjective sleep quality n (%)  Very good  Fairly good  Fairly bad  Very bad | 52 (17.3)  187 (62.1)  60 (19.9)  2 (0.7) | 30 (21.9)  84 (61.3)  22 (16.1)  1 (0.7) | 86 (11.9)  479 (66.5)  137 (19.0)  18 (2.5) | 7 (6.7)  75 (72.1)  19 (18.3)  3 (2.9) |
| Score sleep latency (0-4) \|  M (SD)  Pairwise comparison via  *Kruskall Wallis test* ^a^  Dresden  Austria  Hungary  Japan | 1.20 (0.9)  -  *p>*.99  *p>*.99  *p>*.99 | 1.23 (0.8)  *p>*.99  -  *p>*.99  *p=.*17 | 1.23 (0.9)  *p>*.99  *p>*.99  -  *p=.*049 | 0.98 (0.8)  *p>*.99  *p=.*15  *p=.*049  - |
| Sleep latency in min M (SD) | 21.45 (19.2) | 19.06 (15.8) | 22.90 (25.0) | 19.00 (25.5) |
|  |  | | | |
| Pairwise comparison via  *Kruskal-Wallis test* ^a^  Germany  Austria  Hungary  Japan | -  *p>*.99  *p>*.99  *p=.*06 | *p>*.99  -  *p>*.99  *p=.*79 | *p>*.99  *p>*.99  -  *p=.*04 | *p=.*06  *p=.*79  *p=.*04  - |
| Sleep latency n (%)  < 15  16-30  31-60  > 60 minutes | 61 (20.3)  145 (48.3)  70 (23.3)  25 (8.3) | 22 (16.1)  72 (52.6)  33 (24.1)  10 (7.3) | 157 (21.8)  315 (43.8)  175 (24.3)  73 (10.1) | 32 (30.8)  47 (45.2)  20 (19.2)  5 (4.8) |
| Score sleep duration (0-4) \|M (SD)  Pairwise comparison via  *Kruskal-Wallis test* ^a^  Germany  Austria  Hungary  Japan | 0.31 (0.6)  -  *p>*.99  *p=.*07  *p=.*03 | 0.21 (0.5)  *p>*.99  -  *p=.*004  *p=.*003 | 0.42 (0.7)  *p=.*07  *p=.*004  -  *p>*.99 | 0.45 (0.6)  *p=.*03  *p=.*003  *p>*.99 |
| Sleep duration in min (SD)  Pairwise comparison via  *Kruskal Wallis test* ^a^  Germany  Austria  Hungary  Japan | 7.33 (1.2)  -  *p>*.99  *p<*.001  *p<*.001 | 7.34 (0.9)  *p>*.99  -  *p<*.001  *p<*.001 | 7.00 (1.1)  *p<*.001  *p<*.001  -  *p>*.99 | 6.90 (1.0)  *p<*.001  *p<*.001  *p>*.99  - |
| Sleep duration n (%)  > 7 hours  6-7 hours  5-6 hours  < 5 hours | 228 (75.7)  54 (17.9)  17 (5.6)  2 (0.7) | 112 (81.8)  21 (15.3)  4 (2.9)  - | 490 (68.1)  166 (23.1)  54 (7.5)  10 (1.4) | 62 (59.6)  37 (35.6)  5 (4.8)  - |
| Score sleep efficiency (0-4) \| M (SD)  Pairwise comparison via  *Kruskal Wallis test* ^a^  Germany  Austria  Hungary  Japan | 0.47 (0.7)  -  *p>*.99  *p=.*01  *p<*.001 | 0.36 (0.6)  *p>*.99  -  *p>*.99  *p<*.001 | 0.33 (0.6)  *p=.*01  *p>*.99  -  *P* <.001 | 0.08 (0.3)  *p<*.001  *p<*.001  *p<*.001 |
| Sleep efficiency n (%)  > 85%  75-84%  65-74%  < 65% | 191 (63.5)  85 (28.2  20 (6.6)  5 (1.7) | 95 (69.3)  34 (24.8)  8 (5.8)  - | 525 (72.9)  155 (21.5)  38 (5.3)  2 (0.3) | 95 (92.3)  8 (7.7)  -  - |
| Score sleep disturbance (0-4) \|  M (SD)  Pairwise comparison via  *Kruskal Wallis test* ^a^  Germany  Austria  Hungary  Japan | 1.04 (0.4)  -  *p>*.99  *p>*.99  *p<*.001 | 1.04 (0.4)  *p>*.99  -  *p>*.99  *p<*.001 | 1.01 (0.4)  *p>*.99  *p>*.99  -  *p<*.001 | 0.8 (0.5)  *p<*.001  *p<*.001  *p<*.001  - |
| Score use of sleep medication (0-4) \|M (SD)  Pairwise comparison via  *Kruskal Wallis test* ^a^  Germany  Austria  Hungary  Japan | 0.06 (0.3)  -  *p>*.99  *p>*.99  *p>*.99 | 0.08 (0.3)  *p>*.99  -  *p>*.99  *p>*.99 | 0.1 (0.4)  *p>*.99  *p>*.99  -  *p=.*80 | 0.05 (0.3)  *p>*.99  *p>*.99  *p=.*80  - |
| Score daytime dysfunction (0-4) \|  M (SD)  Pairwise comparison via  *Kruskal Wallis test* ^a^  Germany  Austria  Hungary  Japan | 1.00 (0.7)  -  *p>*.99  *p=.*85  *p=.*27 | 0.92 (0.6)  *p>*.99  -  *p=.*14  *p>*.99 | 1.06 (0.6)  *p=.*85  *p=.*14  -  *p=.*01 | 0.85 (0.6)  *p=.*27  *p>*.99  *p=.*01  - |
| M: mean value, SD: standard deviation, ^a^: Bonferoni-corrected significance, in min: in minutes, in hrs: hours, | | | | |
