# Supplementary material for: Nighttime screen use, sleep quality, and smartphone addiction symptoms among medical students: an international cross-sectional study
Source: Front Psychiatry. 2026 Feb 6;17:1735186. doi: 10.3389/fpsyt.2026.1735186 (PMC12920586; doi:10.3389/fpsyt.2026.1735186)
Supplement: Supplementary file 8 [file Supplementaryfile8.docx]

| Supplementary 8:  7.1 Correlation matrix PSQI components & night-time screen use \| Germany | | | | |
| --- | --- | --- | --- | --- |
|  | Screen time before bedtime | Time between the end of screen use and bedtime | Screen use after waking up at night | Disturbance of sleep by an electronic device with a screen |
| PSQI Total | τ=0.135  *p=*.02 | τ=0.070  *p=*.11 | **τ=0.172**  ***p<*.001** | **τ=0.254**  ***p<*.001** |
| Subjective sleep quality | **τ=0.112**  ***p<*.02** | τ=0.067  *p=*.16 | **τ=0.153**  ***p=*.003** | **τ=0.206**  ***p<*.001** |
| Sleep latency | **τ=0.153**  ***p<*.001** | **τ=0.144**  ***p=*.002** | **τ=0.151**  ***p=*.003** | **τ=0.169**  ***p<*.001** |
| Sleep duration | **τ=0.120**  ***p=*.01** | τ=0.083  *p=*.092 | **τ=0.141**  ***p=*.007** | **τ=0.280**  ***p<*.001** |
| Sleep efficiency | τ=0.086  *p=*.08 | τ=0.042  *p=*.39 | **τ=0.127**  ***p=*.02** | τ=0.082  *p=*.16 |
| Sleep disturbance | τ=0.026  *p=*.60 | τ=-0.006  *p=*.91 | **τ=0.124**  ***p=*.02** | **τ=0.269**  ***p<*.001** |
| Use of sleep medication | τ=0.064  *p=*.20 | τ=-0.032  *p=*.53 | τ=0.027  *p=*.61 | **τ=0.216**  ***p<*.001** |
| Daytime dysfunction | **τ=0.105**  ***p=*.03** | τ=-0.085  *p=*.07 | τ=0.066  *p=*.21 | **τ=0.182**  ***p<*.001** |
| Correlation measure: Kendals-Tau-B | | | | |

| 7.2 Correlation matrix PSQI components & night-time screen use \| Austria | | | | |
| --- | --- | --- | --- | --- |
|  | Screen time before bedtime | Time between the end of screen use and bedtime | Screen use after waking up at night | Disturbance of sleep by an electronic device with a screen |
| PSQI Total | τ=0.037  *p=*.60 | τ=-0.036  *p=*.58 | **τ=0.185**  ***p=* .008** | τ=0.135  *p=* .07 |
| Subjective sleep quality | τ=0.009  *p=*.90 | τ=0.078  *p=*.28 | τ=0.075  *p=*.33 | τ=0.092  *p=*0.26 |
| Sleep latency | τ=-0.047  *p=*.50 | τ=-0.028  *p=*.69 | τ=-0.066  *p=*.38 | τ=0.018  *p=*0.83 |
| Sleep duration | τ=0.002  *p=*.98 | τ=0.081  *p=*.27 | **τ=0.216**  ***p=*.006** | **τ=0.214**  ***p=* .01** |
| Sleep efficiency | τ=-0.021  *p=*.78 | τ=-0.046  *p=*.54 | **τ=0.223**  ***p=*.004** | τ=0.033  *p=*.69 |
| Sleep disturbance | τ=-0.056  *p=*.44 | τ=0.079  *p=*.29 | **τ=0.174**  ***p=*.03** | τ=0.104  *p=*.21 |
| Use of sleep medication | τ=0.084  *p=*.26 | τ=-0.037  *p=*.62 | τ=-0.070  *p=*.37 | τ=0.063  *p=*.45 |
| Daytime dysfunction | **τ=0.146**  ***p=*.04** | τ=-0.016  *p=*.82 | **τ=0.178**  ***p=*.02** | **τ=0.204**  ***p=*.01** |
| Correlation measure: Kendals-Tau-B | | | | |

| 7.3 Correlation matrix PSQI components & night-time screen use \| Hungary | | | | |
| --- | --- | --- | --- | --- |
|  | Screen time before bedtime | Time between the end of screen use and bedtime | Screen use after waking up at night | Disturbance of sleep by an electronic device with a screen |
| PSQI Total | τ=0.042  *p=*.14 | τ=0.024  *p=*.40 | **τ=0.154**  ***p=*.008** | **τ=0.144**  ***p<*.001** |
| Subjective sleep quality | τ=0.055  *p=*.08 | τ=0.012  *p=*.70 | τ=0.089  *p=*.008 | τ=0.092  *p=*0.26 |
| Sleep latency | **τ=0.102**  ***p<*.001** | τ=0.059  *p=*.048 | **τ=0.104**  ***p=*.001** | τ=0.069  *p=*.04 |
| Sleep duration | τ=-0.021  *p=*.50 | τ=-0.009  *p=*.78 | τ=0.088  *p=*.009 | **τ=0.214**  ***p=*.001** |
| Sleep efficiency | τ=0.013  *p=*.68 | τ=0.011  *p=*.72 | τ=0.097  *p=*.004 | τ=0.033  *p=*.69 |
| Sleep disturbance | τ=-0.017  *p=*.58 | τ=0.025  *p=*.42 | **τ=0.215**  ***p<*.001** | τ=0.104  *p=*.21 |
| Use of sleep medication | τ=0.056  *p=*.08 | **τ=0.105**  ***p=*.001** | τ=0.055  *p=*.11 | τ=0.063  *p=*.45 |
| Daytime dysfunction | τ=-0.048  *p=*.12 | τ=-0.065  *p=*.03 | τ=0.091  *p=*.006 | **τ=0.204**  ***p=* .01** |
| Correlation measure: Kendals-Tau-B | | | | |

| 7.4 Correlation matrix PSQI components & night-time screen use \| Japan | | | | |
| --- | --- | --- | --- | --- |
|  | Screen time before bedtime | Time between the end of screen use and bedtime | Screen use after waking up at night | Disturbance of sleep by an electronic device with a screen |
| PSQI Total | **τ=0.158**  ***p=*.04** | τ=0.065  *p=*.40 | **τ=0.223**  ***p=*.007** | τ=0.129  *p=*.13 |
| Subjective sleep quality | **τ=0.183**  ***p=*.03** | τ=0.022  *p=*.80 | **τ=0.206**  ***p=*.03** | τ=0.096  *p=*.31 |
| Sleep latency | **τ=0.178**  ***p=*.03** | τ=0.109  *p=*.19 | **τ=0.178**  ***p=*.0046** | τ=0.076  *p=*.41 |
| Sleep duration | τ=-0.112  *p=*0.2 | τ=0.087  *p=*.32 | τ=-0.026  *p=*.78 | τ=0.004  *p=*.97 |
| Sleep efficiency | τ=0.127  *p=*.15 | τ=-0.129  *p=*.14 | τ=-0.012  *p=*.90 | τ=-0.018  *p=*.86 |
| Sleep disturbance | τ=-0.006  *p=*.94 | τ=0.088  *p=*.31 | **τ=0.229**  ***p=*.02** | τ=0.085  *p=*.38 |
| Use of sleep medication | τ=0.058  *p=*.51 | **τ=-0.173**  ***p=*.005** | τ=-0.092  *p=*.33 | τ=-0.070  *p=*.47 |
| Daytime dysfunction | τ=0.115  *p=*.16 | τ=-0.057  *p=*.51 | τ=0.141  *p=*.12 | τ=0.097  *p=*.30 |
| Correlation measure: Kendals-Tau-B | | | | |
