# Supplementary material for: Nighttime screen use, sleep quality, and smartphone addiction symptoms among medical students: an international cross-sectional study
Source: Front Psychiatry. 2026 Feb 6;17:1735186. doi: 10.3389/fpsyt.2026.1735186 (PMC12920586; doi:10.3389/fpsyt.2026.1735186)
Supplement: Supplementary file 9 [file Supplementaryfile9.docx]

| Supplementary 9  8.1 Correlation matrix PSU & night-time screen use \| Germany | | | | |
| --- | --- | --- | --- | --- |
|  | Screen time before bedtime | Time between the end of screen use and bedtime | Screen use after waking up at night | Disturbance of sleep by an electronic device with a screen |
| PSU | τ=0.011  *p=*.79 | **τ=-0.148**  ***p<*.001** | **τ=0.186**  ***p<*.001** | τ=0.076  *p=*.11 |
| Correlation measure: Kendals-Tau-B | | | | |

| 8.2 Correlation matrix PSU & night-time screen use \| Austria | | | | |
| --- | --- | --- | --- | --- |
|  | Screen time before bedtime | Time between the end of screen use and bedtime | Screen use after waking up at night | Disturbance of sleep by an electronic device with a screen |
| PSU | τ=0.053  *p=*.40 | τ=-0.097  *p=*.12 | **τ=0.149**  ***p=*.025** | **τ=0.232**  ***p<*.001** |
| Correlation measure: Kendals-Tau-B | | | | |

| 8.3 Correlation matrix PSU & night-time screen use \| Hungary | | | | |
| --- | --- | --- | --- | --- |
|  | Screen time before bedtime | Time between the end of screen use and bedtime | Screen use after waking up at night | Disturbance of sleep by an electronic device with a screen |
| PSU | τ=0.015  *p=*.57 | **τ=-0.158**  ***p<*.001** | **τ=0.193**  ***p<*.001** | **τ=0.155**  ***p<*.001** |
| Correlation measure: Kendals-Tau-B | | | | |

| 8.4 Correlation matrix PSU & night-time screen use \| Japan | | | | |
| --- | --- | --- | --- | --- |
|  | Screen time before bedtime | Time between the end of screen use and bedtime | Screen use after waking up at night | Disturbance of sleep by an electronic device with a screen |
| PSU | τ=0.035  *p=*.63 | τ=-0.072  *p=*.33 | τ=0.096  *p=*.26 | τ=0.103  *p=*.20 |
| Correlation measure: Kendals-Tau-B | | | | |
