# Supplementary material for: Nighttime screen use, sleep quality, and smartphone addiction symptoms among medical students: an international cross-sectional study
Source: Front Psychiatry. 2026 Feb 6;17:1735186. doi: 10.3389/fpsyt.2026.1735186 (PMC12920586; doi:10.3389/fpsyt.2026.1735186)
Supplement: Supplementary file 10 [file Supplementaryfile10.docx]

Supplementary 10: Generalized Variance Inflation Factor – Predictors for Logistic Regression

| Predictor | GVIF | Df | Adjusted GVIF |
| --- | --- | --- | --- |
| Study site | 1.462228 | 3 | 1.065375 |
| Age | 1.459658 | 1 | 1.208163 |
| Gender | 1.076319 | 2 | 1.018557 |
| PSU | 1.140401 | 1 | 1.067896 |
| Screen time before bed | 1.126481 | 3 | 1.020048 |
| Time between the end of screen use & bedtime | 1.098699 | 3 | 1.015812 |
| Screen use after waking up | 1.217865 | 2 | 1.050509 |
| Disturbance of sleep by device | 1.239092 | 2 | 1.055057 |
| Study period | 1.393573 | 1 | 1.180497 |
| Committed relationship | 1.112742 | 1 | 1.054866 |
| Housing situation | 1.167694 | 1 | 1.080599 |
| Physical activity | 1.037150 | 1 | 1.018406 |
| Financial situation | 1.058350 | 1 | 1.028761 |
| Adjusted GVIF: GVIF^(1/(2*Df)) | | | |
